# How AI and Robotics Will Advance Interventional Radiology: Narrative Review and Future Perspectives

**DOI:** 10.3390/diagnostics14131393

**Published:** 2024-06-29

**Authors:** Jiaming Zhang, Jiayi Fang, Yanneng Xu, Guangyan Si

**Affiliations:** 1 Department of Radiology, Clinical Medical College, Southwest Medical University, Luzhou 646699, China; 20210299120053@stu.swmu.edu.cn (J.Z.); 20220299120060@stu.swmu.edu.cn (J.F.); 2 Department of Radiology, Affiliated Traditional Chinese Medicine Hospital, Southwest Medical University, Luzhou 646699, China; xyneng@swmu.edu.cn

**Keywords:** artificial intelligence, robot, deep learn, machine learning, convolutional neural networks, interventional oncology, interventional neuroradiology, interventional cardiology

## Abstract

The rapid advancement of artificial intelligence (AI) and robotics has led to significant progress in various medical fields including interventional radiology (IR). This review focuses on the research progress and applications of AI and robotics in IR, including deep learning (DL), machine learning (ML), and convolutional neural networks (CNNs) across specialties such as oncology, neurology, and cardiology, aiming to explore potential directions in future interventional treatments. To ensure the breadth and depth of this review, we implemented a systematic literature search strategy, selecting research published within the last five years. We conducted searches in databases such as PubMed and Google Scholar to find relevant literature. Special emphasis was placed on selecting large-scale studies to ensure the comprehensiveness and reliability of the results. This review summarizes the latest research directions and developments, ultimately analyzing their corresponding potential and limitations. It furnishes essential information and insights for researchers, clinicians, and policymakers, potentially propelling advancements and innovations within the domains of AI and IR. Finally, our findings indicate that although AI and robotics technologies are not yet widely applied in clinical settings, they are evolving across multiple aspects and are expected to significantly improve the processes and efficacy of interventional treatments.

## 1. Introduction

With the rapid development of technology, the applications of artificial intelligence (AI) and robotics in the medical field are becoming increasingly widespread, especially in interventional radiology (IR) where significant progress has been made [1,2]. Initially named “interventional diagnostic radiology” by Dr. Alexander R. Margulis in 1967 [3], the concept of IR was systematically elaborated by the academic Wallace as early as 1976 and refers to fluoroscopically guided operative techniques for diagnosis and treatment [4]. The area of interest is visualized by image enhancement combined with percutaneous puncture and angiography. Developments in medical precision therapy and technological advances in the manufacture of medical devices have contributed to the rapid growth of the field. IR is a minimally invasive treatment with minimal trauma, precise efficacy, and few complications. Furthermore, interventional therapy not only reduces the need for expensive operating suites and general anesthesia but also greatly reduces the recovery time of patients with the smallest possible surgical wound, so it has been widely used in clinical treatment [5]. Although the point of intervention may be small, the effect of the intervention may be as profound as that of major surgery. Commonly used interventional diagnosis and treatment methods in clinical practice include image-guided needle biopsy of various organs, tumor ablation, diagnosis and treatment of cardiovascular and cerebrovascular diseases, etc. The continuous advancement of IR has reduced the cost of medical care and made the treatment effect more obvious. Replacing many traditional open surgical procedures, it can play an increasing role in precision medicine and value-driven healthcare [1,2,3].

In recent years, the explosive development of AI has also promoted the rapid progress of IR, and AI has made significant contributions to solving various biomedical problems. As a subset of computer science, AI aims to emulate human cognitive functions, offering smarter, efficient, and innovative solutions through learning and problem-solving [6,7]. A primary strength of AI lies in its capability to harness vast multi-parametric data, extracting invaluable insights for patient-specific clinical decisions [6,8]. Among the myriad AI learning techniques, machine learning (ML) and deep learning (DL) stand out in medical applications [9]. ML is versatile, covering techniques from simple linear regression to complex ensemble methods. DL, a subset of ML, is inspired by the structure of the human brain and uses layers of algorithms called neural networks. It excels in recognizing patterns in unstructured data like images and audio. In the medical field, AI techniques have been successfully applied to assist in diagnosis, disease prediction, and personalized treatment, providing powerful technical support for physicians. AI-assisted diagnostics can perform the initial lesion screening and quickly extract valuable diagnostic-related information from massive data [10,11] while avoiding the subjectivity differences of manual image reading. AI-assisted image processing algorithms have sophisticated image segmentation and alignment functions that provide accurate information about lesion structure [12,13]. Due to its assistance, interventional therapy can reduce radiation exposure for physicians and patients while enhancing therapeutic effects, and it is increasingly embraced by physicians in clinical practice [14].

At the same time, robotics has also been widely applied in the medical field. Medical robots have demonstrated outstanding potential in operation navigation, rehabilitation therapy, as well as remote diagnosis and treatment [15]. In interventional therapy, robotic-assisted operation systems have become an essential method, with advantages such as precision, stability, and repeatability, thus improving operational outcomes and reducing the risk of intraoperative complications. The combination of AI and robotics in interventional therapy has broad prospects for development. AI techniques can provide more accurate predictions and plans for operation by analyzing massive medical data while providing real-time feedback to the robot to improve treatment outcomes. In this context, researchers are exploring ways to combine AI techniques with robotic-assisted interventional therapy to achieve safer and more efficient interventional treatment.

Starting with advances in interventional therapy made with AI techniques, we explore the role of AI techniques in interventional procedure planning and execution, as well as the advantages of robots in achieving precise operations and reducing intra-operative risks. Moreover, we discuss the future trends and potential applications in AI, robotics, and interventional therapy. Our literature search was conducted using major databases, including PubMed, Scopus, Web of Science, and IEEE Xplore, targeting publications from March 2019 to March 2024. We focused on articles written in English as well as those with larger sample sizes to ensure the reliability and accessibility of the data. To ensure the accuracy and relevance of our review, the literature primarily sourced for this study was identified through a search using following strategy: 1. (“Artificial Intelligence” OR “AI” OR “Machine Learning” OR “Neural Networks” OR “Robotics” OR “Robots” OR “Autonomous Systems” OR “Automation”) AND (“Endovascular Therapy” OR “Interventional Radiology”); 2. (“Artificial Intelligence” OR “AI” OR “Machine Learning” OR “Neural Networks” OR “Robotics” OR “Robots” OR “Autonomous Systems” OR “Automation”) AND (“Coiling” OR “Intracranial Aneurysms” OR “Acute Ischemic Stroke” OR “Neurointerventional Radiology”); 3. (“Artificial Intelligence” OR “AI” OR “Machine Learning” OR “Neural Networks” OR “Robotics” OR “Robots” OR “Autonomous Systems” OR “Automation”) AND (“Interventional Oncology” OR “Tumor Ablation” OR “Transarterial Chemoembolization”); 4. (“Artificial Intelligence” OR “AI” OR “Machine Learning” OR “Neural Networks” OR “Robotics” OR “Robots” OR “Autonomous Systems” OR “Automation”) AND (“Cardiovascular Intervention” OR “Coronary Angioplasty” OR “Percutaneous Coronary Intervention” OR “Valvuloplasty” OR “Stent Placement”). By employing this strategy, we have ensured that the most influential and recent studies are covered. Finally, this review describes the limitations and ethical concerns when applying these techniques in interventional radiology. As technology advances, we look forward to AI and robotics technologies playing a more prominent role in interventional therapy, resulting in better patient treatment outcomes and quality of life.

## 2. Application of AI in Interventional Therapy

### 2.1. Interventional Oncology

In recent years, the application of AI in medical research has been continuously deepening, and its use in the field of oncology has become one of the current hotspots. AI has suitable applications in assisting tumor diagnosis and differential diagnosis, tumor grading, prognosis evaluation, and preoperative prediction [16]. In tumor treatment, IR methods mainly include chemoembolization infusion and tumor ablation. Combining AI techniques to optimize the intervention process, especially in hepatocellular carcinoma (HCC), can help ensure the effectiveness of lesion embolization and reduce postoperative reactions in patients.

Interventional therapy is commonly used in patients with intermediate to advanced liver cancer, and transarterial chemoembolization (TACE) is currently recognized as one of the most used non-surgical treatments for liver cancer. Although directly applying AI to TACE treatment still has significant challenges, it can help develop individualized treating plans for liver cancer patients and provide a predictive reference for the effects of interventional therapy and the later progression of tumors. In the TACE treatment of advanced HCC, Lu Zhang et al. used DL to combine clinical variables and post-processed digital subtraction angiography (DSA) image information from patients with hepatocellular carcinoma prior to TACE treatment to build the model [17]. The results show that the AUC of the TACE treatment response prediction model exceeded 80% for both the internal validation cohort and the external validation cohort, and the DSA-NET model can identify patients who respond effectively to TACE treatment, making it a promising tool for predicting TACE efficacy. Similarly, Abajian A et al. attempted to develop an ML model using MR imaging and qEASL (quantitative European Association for the Study of the Liver) criteria to predict whether patients are treatment responders or non-responders and achieved an overall accuracy of 78% [18]. Patients unsuitable for TACE may experience extrahepatic spread or vascular invasion progression after initial monotherapy with TACE [19]. Zhicheng Jin et al. designed a model combining clinical radiological and radiomic features to predict the development of extrahepatic metastasis or vascular invasion in non-advanced HCC patients following TACE monotherapy. In this multicenter study, the AUC for the combined model training set was 0.911, and it was 0.847 in the test set, thus demonstrating relatively high sensitivity and specificity [20].

The combination of radiomics and DL techniques can improve the performance of diagnostic models. Dan Liu et al. [21] retrospectively included ultrasound imaging data from 130 patients with HCC and constructed and validated three models for predicting the response to TACE. The models were a DL radiomics-based contrast-enhanced ultrasound (R-DLCEUS) model, an ML radiomics-based time-intensity curve of contrast-enhanced ultrasound (R-TIC) model, and an ML radiomics-based B-mode image (R-BMode) model. The models were compared using the area under the receiver operating characteristic curve, and the results showed that R-DLCEUS could predict the response to TACE after the first treatment session with convenience and accuracy and had significant differences compared to R-TIC (*p* = 0.034) and R-BMode (*p* = 0.039).

Thermal ablation is a minimally invasive procedure for treating small or unresectable tumors. Microwave ablation and radiofrequency ablation have the advantages of shorter operation time, less bleeding, and fewer complications [22]. Accurate lesion targeting is one of the keys to successful ablation, and utilizing DL/ML method to align and integrate preoperative and intraoperative images for lesion localization and recognition of the ablation area is a promising direction. In liver tumor ablation, simple 2D ultrasound guidance may not be sufficient for accurate puncture and assessment of lesion ablation effects because of the poor image quality and low soft tissue contrast [23], and preoperative scanning 3D images are needed to provide better reference. Wei et al. employed DL methods for image registration, facilitating real-time estimation of ultrasound image planes in 3D CT/MR, which has the potential to improve the robustness and precision of intraoperative patient registration [24]. However, the current accuracy of this method is greatly affected by the vascular structures present in the ultrasound images and the ultrasound section’s angle. Accurate ablation margin (AM) assessment during therapy will provide important information to the operating physician to improve ablation outcomes. And researcher Chao An used DL-based deformable image registration (DIR) technology to evaluate the AM of HCC patients after MWA in a study of 141 HCC patients [25]. The results showed that the AUC value of the DL-based registration technology was higher than that of the traditional registration technology, and the measured AM was more accurate compared to current methods. When confronting the ablation of large lesions, physicians often need to perform multiple and overlapping ablations to ensure complete eradication of the lesion while minimizing damage to adjacent normal tissues. Preoperative planning plays a crucial role in Radiofrequency Ablation (RFA) treatment. However, incorporating constraints of nearby structures into the preoperative planning presents a challenging task. Liang et al. have proposed an automated RFA planning method. This method generates RFA plans that are theoretically feasible and have demonstrated minimal use of RFA electrodes, complete tumor coverage, and minimal ablation of normal tissue during validation [26].

In summary, from the recognition and diagnosis of tumors in medical imaging, through intraoperative guidance, to postoperative assessment in cancer treatment, AI technology has been integrated into every phase of tumor intervention. These applications hold the promise of enhancing the precision and efficiency of treatments. As AI technology continues to advance, the future will likely see more intelligent medical decision-making and treatment plans become a reality.

### 2.2. Interventional Neuroradiology

Cerebrovascular diseases (CVD) are one of the leading causes of death and disability worldwide, characterized by high incidence, long disease course, and high rates of disability and mortality. Neurointerventional techniques have become a critical approach in treating cerebrovascular diseases [27]. Neuro-intervention aims to alleviate patient suffering, shorten recovery time, and improve treatment outcomes by offering minimally invasive diagnostic and therapeutic procedures for cerebrovascular diseases. However, neurointerventional therapies are highly complex, requiring physicians to have extensive experience and sophisticated skills to identify lesions and plan operating approaches accurately.

The development of AI techniques is a new tool for interventional physicians, playing a significant role in precision diagnosis, risk stratification, and prognostic assessment of CVD. In addition, integrating advanced AI techniques with clinical data and imaging information can provide more structural and functional details on CVD, which is crucial in the interventional diagnosis and treatment of CVD [28,29]. This section briefly discusses the application of AI technology in cerebrovascular diseases and summarizes the research methods and key points of these technologies in Table 1.

#### 2.2.1. Cerebral Angiography

Cerebral angiography is an important diagnostic tool for detecting cerebrovascular diseases, including cerebral aneurysms, cerebral hemorrhage, large-vessel occlusion (LVO), and other vascular lesions. Compared to computed tomography angiography (CTA) and magnetic resonance angiography (MRA), DSA features high-resolution images and the ability to display blood flow dynamics in real-time, offering clearer visualization of complex vascular structures and lesions. Image registration technology is key in medical image analysis, providing the prerequisites for medical image fusion, segmentation, reconstruction, and comparison. By fusing preoperative 3D images with intraoperative 2D images, advanced image registration techniques using ML make virtual angiography a valuable tool for interventional physicians to formulate treatment plans [30,31]. In addition, to provide better DSA diagnostic results, various image processing methods are used to remove motion artifacts in DSA images.

To reduce artifacts caused by subtracting real-time images from mask images under DSA perspective and motion artifacts caused by patient movement, thus avoiding unnecessary radiation exposure, Yufeng Gao et al. adopted a deep learning-based Residual Density Block (RDB) algorithm and trained the model using a supervised generative adversarial network strategy [32]. In conclusion, although the authors show that the model effectively reduces the artifacts mentioned before, it did not indicate whether the model output has misregistration issues when there are misregistration artifacts present in DSA images.

Following this, Daiju Ueda et al. proposed a deep learning-based brain angiography model without misregistration artifacts [33]. This model does not require subtraction of mask images but generates DSA-like images by extracting features only from dynamic angiography to remove non-vascular backgrounds. Experimental results show that the angiography generated by the deep learning-based model is not only comparable to DSA images but also has no significant misregistration, and it can reduce image artifacts as well. The development of this model provides more valuable brain angiography images for clinical use.

Furthermore, radiation exposure in DSA imaging is a concern for both patients and interventional physicians. Traditional 3D-DSA scanning and reconstruction methods require patients to be continuously exposed to radiation. Huang Xuan Zhao et al. analyzed the reconstructed images of 202 suspected intracranial aneurysm patients and proposed a self-supervised learning method for three-dimensional DSA reconstruction using ultra-sparse two-dimensional projections [31]. Compared to traditional reconstruction methods, they claim that only eight two-dimensional images are needed to efficiently reconstruct multi-scale cerebral vasculature, and patients only need to endure about 1/16 of the radiation dose.

#### 2.2.2. Intracranial Aneurysms

Intracranial aneurysms (IAs) result from local abnormal dilation of the walls of cerebral arteries, usually caused by a weakness in the vessels’ inner lining [34]. They are a major cause of subarachnoid hemorrhage, second only to hypertensive cerebral hemorrhage and thrombosis in incidence. Approximately 80% to 90% of aneurysms are discovered after rupture, as the formation of IAs usually has no apparent symptoms [35]. As the gold standard for diagnosing IA, DSA holds an irreplaceable position in endovascular therapy due to its high image resolution. However, compared to the more established computer-aided diagnosis (CAD) systems for CTA and MRA, the CAD systems based on DSA are still in the research and development stage [36]. Yuwen Zeng et al. proposed a DL method based on spatial information fusion for the automatic diagnosis of intracranial aneurysms in 3D DSA images, aimed at assisting radiologists in image interpretation to avoid missed and incorrect diagnoses [37]. This method embeds spatial contextual information from consecutive frames into a single two-dimensional image, allowing time series with significant inter-frame correlation to be directly trained on a 2D convolutional neural network. This approach achieved a 98.89% accuracy rate, along with 99.38% sensitivity and 98.19% specificity in clinical image validation, providing direction for the development of reliable CAD tools under DSA.

Although many studies have highlighted the potential of AI models in detecting aneurysms and predicting aneurysm ruptures, we must acknowledge that these studies still lack sufficient training data [38]. In research on predicting aneurysm ruptures, we even find instances where the predictive accuracy of AI models does not surpass that of traditional statistical methods. In the study by Nicolás Amigo et al., morphological characterization and fluid dynamics simulations were conducted to classify the rupture status of 71 patient-specific cerebral aneurysms (36 unruptured, 35 ruptured) using an ML approach combined with statistical techniques [39]. The study assessed the importance of eleven morphological parameters and six hemodynamic parameters as predictors of rupture status. Random Forest achieved the best AUC among various classifiers, but its predictive accuracy was still weaker than traditional logistic regression methods. In conclusion, we need a multicenter study with a large sample size to better assess the prospects of AI applications in this area.

In the context of IA interventions, the significance lies not only in the precise positioning and deployment of embolic materials during the procedure but also in the selection of embolic materials and the prevention and management of postoperative complications. Each of these elements influences patient outcomes. The application of AI technologies to assist physicians offers an additional layer of security for the treatment of IA patients.

The endovascular technique of IA treatment involves identifying the lesion location through angiography, selecting appropriate embolic materials to fill the aneurysm through 3D DSA and CTA images, or using flow reduction methods such as pipeline embolization device (PED) to promote intraneurysmal thrombosis and endothelialization, eventually leading to occlusion. For patients treated with PED pipeline embolization devices, IAs are generally occluded within a few months, requiring at least 6 months of follow-up evaluation of occlusion status [40]. Also, the flow-diverter stents can become occluded, leading to thromboembolic and rupture complications [41]. To solve this problem, Some researchers have attempted to use AI technology to predict the occlusion status of aneurysms after embolization. Shiraz Bhurwani et al. first analyzed intraoperative DSA images before and after IA embolization and used a deep neural network (DNN) along with Angiographic Parametric Imaging (API) to predict IA occlusion, demonstrating the feasibility of this approach. Building on this, the following study aimed to avoid X-ray attenuation at the embolization device by capturing changes in blood flow within the carrier artery distal to the IA to predict the occlusion status of IAs within six months after PED deployment; the predictive accuracy also met expectations [42]. These intraoperative predictions can be directly fed back to doctors, assisting them in determining whether additional interventions are necessary. Intraoperative predictions can be directly fed back to doctors, assisting them in determining whether additional interventions are necessary.

Artificial intelligence can also play a crucial role in the decision-making process during IA treatment. By building models based on machine learning or deep learning, AI can provide guidance for the selection of interventional procedures and interventional tools, which may benefit inexperienced physicians. Specifically, in the treatment of aneurysms, S. Fujimura et al. developed machine learning models to predict the size and length of the first coil used in coil embolization therapy [43]. According to this study, while the model’s predictive consistency is lower than that of experienced physicians, it performs better than trainees. Furthermore, this model has the potential to be expanded to assist physicians in selecting the ideal coil.

#### 2.2.3. Acute Ischemic Stroke

The global incidence of Acute Ischemic Stroke (AIS) reaches 7.59 million cases annually, accounting for about 80% of all stroke cases. Rapid and accurate diagnosis of AIS is crucial for providing timely treatment and improving patient prognosis. Particularly in AIS patients with LVO, especially anterior circulation LVOs, endovascular thrombectomy (EVT) using stents or aspiration catheters can be beneficial.

Commercial DL software such as Brainomix’s e-Stroke, United Imaging’s uAI-HematomaCare, Viz AI’s VIZ LVO, and Avicenna.ai’s CINA^®^ system [44,45] can now assist physicians in accelerating the diagnostic and treatment process for stroke patients. This includes various aspects of stroke diagnosis and treatment processes such as detection, classification, segmentation, and prediction of infarctions or hemorrhages.

Recent research by Omer Bagcilar employed a multicenter approach to validate a technology based on DL [46]. This technology utilized nnDetection, an advanced self-configuring 3D object detection network, which demonstrated over 98% accuracy in identifying LVO in an independent external dataset. In contrast to traditional image processing software (iSchemaView, RAPID CTA) that reported numerous false positives due to vascular asymmetry, commercially available DL software (such as Avicenna.ai’s and e-Stroke) along with nnDetection showed higher diagnostic performance, exhibiting relatively high consistency with experienced radiologists [47,48].

Although the reliability and accuracy of these algorithms are currently limited by data quality and quantity, and their application scenarios and limitations need to be carefully evaluated, in the future, more advanced and comprehensive stroke diagnosis and treatment platforms equipped with AI techniques are expected to achieve universal monitoring, providing life-saving protection for patients suffering from such sudden illnesses.

To address the explainability issues of ML models, Islam. N et al. designed and trained an AutoML model to predict endovascular mechanical thrombectomy in acute large vessel ischemic stroke [49]. It compared the model’s performance against various traditional ML methods, predicting patients’ modified Rankin scores (mRS) at discharge and three-month follow-up. The findings revealed that the AutoML model outperformed traditional ML models, with the highest test accuracy for traditional models being 76.5% using the SVM classifier, while AutoML achieved an accuracy of 88.23% with AutoGluon. This AutoML model integrates the SHAP (SHapley Additive exPlanations) framework to explain the importance of each feature, enhancing clinical confidence.

EVT significantly improves the treatment of patients with acute ischemic stroke, but given the variability in physician experience, intraoperative judgment may be compromised, potentially leading to serious consequences [50,51]. Benjamin J. Mittmann et al. proposed deep learning-based methods to identify and classify clots in DSA sequences, reducing the risk of overlooking clots during treatment [52].

**Table 1:** Studies Referenced in the Interventional Neuroradiology Section Applying AI Techniques.

Research	Date	Training Data SET	Method	Aim	Result
H. Zhao et al. [31]	2022	202 cases from different hospitals	Self-supervised Learning	Utilize deep learning to decrease the number of images required for 3D-DSA reconstruction, thereby minimizing radiation	Generated high-quality 3D-DSA from 202 cases
Y. Gao et al. [32]	2019	628 pairs of head data and 690 pairs of leg data	RDB, GAN	Employ deep learning techniques to reduce vascular artifacts	Superior performance in human head and leg tests
D. Ueda et al. [33]	2021	608 sequences from 40 patients	DL Model	Generate vascular silhouettes from dynamic angiography using deep learning methods to avoid motion artifacts	Avg PSNR 40.2 dB, SSIM 0.97
Y. Zeng et al. [37]	2019	300 sequences from 263 patients	SIF Method	Train deep learning models with 2D image sequences for IA recognition in 3D-RA, reducing training costs	98.89% accuracy
N. Amigo et al. [39]	2021	71 sequences from 71 patients	ML Algorithms	Automate cerebral aneurysm rupture status classification	RF with the highest accuracy of 0.75
Shiraz Bhurwani et al. [42]	2022	80 sequences from 80 Patients	DNN	Predict the occlusion status of aneurysms post-embolization using APIs and DNN models	Accuracy 78.6%, AUROC 0.77
S. Fujimura et al. [43]	2023	2377 aneurysms in 2215 patients	ML Algorithms	Implement the first coil size selection for IA embolization using AI during the procedure	The ML model achieved 96.7% and 100% accuracy in predicting the size and length
Islam. N et al. [49]	2023	5110 instances with twelve attributes	AutoML Model	Generate explainable AI models to predict stroke events	The autoML group has a maximum accuracy of 88.23%, better than the traditional ML group
O. Bagcilar et al. [46]	2023	2425 cases from 5 centers	NNDetection	Automate the detection of Large Vessel Occlusion (LVO) and collateral scoring on CTA	98.26% accuracy
B. J. Mittmann et al. [52]	2022	1068 DSA sequences from 260 patients	LSTM, GRU Networks	Automatically distinguish whether thrombectomy in ischemic stroke patients produces new emboli using intraoperative 2D images	Highest MCC 0.77, AUC 0.94

NNDetection: Neural Network Detection. RF: Random Forest. AUROC: Area Under the Receiver Operating Characteristic. SIF: Spatial Information Fusion. PSNR: Peak Signal-to-Noise Ratio. SSIM: Structural Similarity Index. RDB: Residual Dense Block. GAN: Generative Adversarial Network. LSTM: Long Short-Term Memory. GRU: Gated Recurrent Unit. AUC: Area Under Characteristic.

### 2.3. Interventional Cardiology

Cardiovascular disease is currently a serious health threat to human society worldwide, and in recent years, morbidity and mortality rates have been on the rise [53]. Over the past decade, interventional cardiology has made tremendous progress. AI has achieved some advancements in designing interventional treatment strategies for cardiovascular diseases, optimizing procedures, and reducing complications. This section mainly discusses the application of AI technology in cardiovascular diseases and summarizes the research methods and key points of these technologies in Table 2.

#### 2.3.1. Coronary Heart Disease

Coronary heart disease (CHD) is a group of heart diseases caused by coronary artery atherosclerosis, leading to narrowing or blockage of the artery lumen, resulting in myocardial ischemia, hypoxia, or necrosis. As one of the leading causes of death worldwide, CHD causes about 9 million deaths annually, according to data from the World Health Organization. In the process of diagnosing and treating CHD, manually identifying and characterizing coronary artery plaques requires extensive expertise, is time-consuming, and is highly dependent on image quality, which can be easily affected by speckle noise. These challenges have led to the development of Computer-Aided Diagnosis systems designed to automate image processing and identify and characterize coronary artery plaques through invasive or non-invasive imaging techniques. For instance, The AI model used by Miguel Nobre Menezes in a multicenter validation set accurately segments CAG and demonstrates good performance across various performance metrics [54,55]. In stenosis recognition, most studies have confirmed the feasibility of real-time coronary artery stenosis detection, which can effectively support clinical decision-making for interventional therapy [56,57,58,59,60,61]. Recently, Chao Cong et al. showed us a fully automated workflow based on end-to-end DL, which realizes efficient safety screening and precise positioning of narrows, improves training efficiency, and reduces overfitting [60].

In addition, among other invasive coronary imaging methods, IVUS and OCT can visually assess high-risk plaques as well as vascular stenosis [62,63]. These imaging techniques are useful for evaluating vessel size, visualizing post-operative stent expansion, and identifying stent edge dissections and stent malposition. However, IVUS’s frame-by-frame analysis of the entire vascular segment is time-consuming, limiting its application despite the importance of plaque characterization for risk stratification. To address this limitation, Cho et al. developed a DL algorithm to classify attenuated and calcified plaques in IVUS images [64]. This algorithm could enhance the speed of IVUS assessment of high-risk plaques and optimize the identification of attenuated and calcified plaques. For CHD patients requiring percutaneous coronary intervention (PCI), inadequate stent deployment can lead to in-stent restenosis and other complications in treating coronary artery disease. While IVUS is used to optimize PCI after stent placement and prevent inadequate stent deployment [65], pre-procedure estimation of stent expansion and early warnings before deployment can reduce adverse events, identify high-risk lesions in advance, and reduce procedure time and patient radiation exposure. For example, Min et al. designed a DL model based on pre-PCI IVUS images to predict insufficient stent expansion during PCI [66], provide a preoperative reference for physicians, and avoid the need for post-stent-release adjustments. Their theoretical basis is that the postoperative minimal stent area and stent length measured by IVUS are independent predictors of in-stent restenosis [67]. With a classification model based on CNNs and derived features from masked images to predict inadequate stent expansion, the maximum accuracy reached 94%, and the prediction results showed a good correlation with post-IVUS results. These studies indicate the potential directions and feasibility of AI-assisted PCI treatment: preoperative assessment of coronary lesions and risk classification, intraoperative navigation by AI-enhanced intervention robots, and reminders to the operator of potential deficiencies during the operation, reducing postoperative adverse events.

#### 2.3.2. Valvular Heart Disease

Valvular heart disease refers to diseases involving the aortic, mitral, tricuspid, and pulmonary valves, with the incidence increasing with age [68]. It manifests as valve stenosis or insufficiency and is one of the more common chronic heart diseases. Aortic stenosis (AS) is the most acquired valvular heart disease, and transcatheter aortic valve replacement (TAVR) is suitable for symptomatic severe AS patients. Initially, TAVR was suggested as a minimally invasive treatment for patients with high-risk AS, but now TAVR is becoming a promising option for patients with low- to intermediate-risk AS [69]. TAVR equal to or superior to surgical aortic valve replacement has also been demonstrated in patients at intermediate surgical risk [70]. In addition, patients with asymptomatic severe AS with TAVR have been found to have better outcomes than those with moderate AS.

As a screening tool for undiagnosed patients, a simple routine test -AI ECG can successfully identify patients with moderate to severe AS with high performance. Shelly et al. trained ML models using ultrasound electrocardiogram images as input data and used CNNs to realize fully automated detection of AS(AUC 0.85) [71].

In the process of pre-procedural assessment for TAVR, the application of DL has become increasingly evident. Traditionally, evaluators have relied on semi-automated or automated tools to enhance work efficiency. However, due to the complex anatomical structure and pathological variations of the aortic root, manual adjustments are often necessary to ensure the accuracy of these tools. Many methods based on DL, capable of automatically measuring the morphology of the aortic valve, have been limited by the exclusion of certain anatomical variations of the aortic valve, resulting in a lack of robustness in the model. Moyang Wang et al. developed a fully automated algorithm based on DL(FORSSMANN) specifically for the pre-TAVR CT assessment and detection of anatomical risk factors [72]. This multicenter research incorporated a large sample size to enhance the accuracy and robustness of the algorithm, and the results demonstrated that FORSSMANN, in its capability to spatially locate specific anatomical structures, showed strong consistency with senior observers in terms of the Euclidean distance, both in the internal validation dataset (452 cases) and the external validation dataset (100 cases). Additionally, FORSSMANN outperformed junior observers in detecting five anatomical risk factors.

Moreover, despite advances in TAVR technology, cerebrovascular events (CVEs) remain one of the most dreaded complications, and their multifactorial origins are difficult to fully explain using clinical predictive factors. Traditional statistical methods like logistic regression that incorporate a few independent risk factors, such as atherosclerosis, atrial fibrillation, balloon dilation, and device dislocation, cannot fully explain the occurrence of CVEs. In [73], Taishi Okuno et al. proposed using DL to develop a predictive model for CVEs after TAVR [74], selecting a rare event autoencoder as the predictive model, with an AUC of 0.79 (0.65–0.93). The DNN approach used in this model can address the limitations of traditional single-variable and multi-variable regression techniques for predicting serious but rare complications like CVEs to some extent. Similar studies, such as the one by Oliver Maier [73], also indicate that DL methods can generate more accurate risk models for predicting CVE. Furthermore, high-degree atrioventricular block is also one of the common complications after TAVR, and AI also has a high predictive value in the PPI caused by it. Vien T. Truong et al. analyzed 557 sinus rhythm patients who underwent TAVR for AS and compared ML methods and logistic regression for predicting PPI risk after TAVR [75]. The results showed that the random forest (RF) model incorporating post-TAVR electrocardiogram data (AUC 0.81) more accurately predicted PPI risk compared to the RF model without TAVR ECG data (AUC 0.72). Similarly, Takahiro Tsushima et al.’s [76] study with a larger patient sample also demonstrated that ML algorithms could accurately predict the risk of PPI implantation after TAVR. According to the research conducted by these scholars, ML has the potential to improve patient selection and risk management in interventional cardiovascular procedures, and compared to traditional logistic regression analysis, it makes better predictions.

**Table 2:** Studies Referenced in the Interventional Cardiology Section Applying AI Techniques.

Research	Date	Training Data SET	Method	Aim	Result
Cong C. et al. [60]	2023	2161 videos from 230 participants	DL	Train auto CAG model to reduce the burden on high-capacity medical centers	Accuracy 0.85, sensitivity 0.96, AUC for LCA and RCA 0.68 and 0.70
Miguel Nobre Menezes [54,55]	2023	117 cases from 90 patients	ML and DL	Using multi-center data to validate the feasibility and reliability of automatic segmentation of CAG images	Results are similar to those from single-center studies, with an accuracy of 99.9%
Cho et al. [64]	2021	498 IVUS image sets from 498 patients	DL(CNN)	Train models using IVUS to identify high-risk coronary artery lesions.	Ensemble model sensitivity 80%, specificity 96%, overall accuracy 93%
Min et al. [66]	2021	28,952 frames from 515 patients	DL (CNN) and XGBoost	Train IVUS models to predict events of inadequate coronary stent expansion	Accuracy 94%, AUC 0.94
Michal Cohen-Shelly et al. [71]	2021	129,788 cases	DL	Use AI-ECG to detect asymptomatic AS, aiding in early discovery and treatment	AUC 0.85, sensitivity 78%, specificity 74%
Moyang Wang et al. [72]	2023	230,486 images from 800 candidates	DL	Build models for pre-TAVR CT evaluation and anatomical risk factor detection	High correlation with senior observers, ICC up to 0.998
Taishi Okuno et al. [74]	2021	Clinical and MDCT data from 1492 patients	DL Auto-encoder	Train models using combined clinical and radiological features to predict CVE after TAVR	AUC 0.79
Vien T. et al. [75]	2023	Data from 557 patients	ML	Train and evaluate ML models to assess the risk of PPI after TAVR and compare with traditional logistic regression models	RF model AUC 0.81, better than logistic regression model AUC 0.69

AUC: Area Under Characteristic. CAG: Coronary Angiography. IVUS: Intravascular Ultrasound. ECG: Electrocardiogram. PPI: Permanent Pacemaker Implantation. TAVR: Transcatheter Aortic Valve Replacement. MDCT: Multi-Detector Computed Tomography.

### 2.4. Development Directions of Interventional AI Application

The effectiveness of interventional therapy heavily depends on physicians’ profound understanding of both the equipment and human anatomy, as well as their interactions, which are difficult to fully express through written text. In this context, simulators offer a relatively inexpensive and controllable training method. In the field of neurosurgery, a systematic review summarizing the role of simulators found that, based on 26 included studies, most concluded that practitioners benefit from simulation in aneurysm clipping operations [77]. However, limitations such as the poor storage and low fidelity of cadaver models, the lack of tactile feedback in VR models, and the poor reusability of 3D printed models have hindered the widespread adoption of simulators.

Conversely, given the enclosed design characteristics of intervention simulators and the advancements in computer technology, commercial simulators now possess decent simulation capabilities and have made progress in terms of fidelity, reusability, and portability. In the field of endovascular therapy, commercial vascular simulators like ANGIO MENTOR^®^ and Mentice VIST^®^ effectively mimic various operations of guidewires and catheters within vessels, providing realistic tactile feedback and high-quality interventional procedure simulations. These simulators are not only used for training across various types of interventional surgeries but, coupled with appropriate software, can also create 3D models of individual patients’ actual lesion structures using AI technology to replicate and register images of real vascular conditions accurately. On this basis, force feedback simulations built on deep learning and real-time blood flow simulations developed with computational fluid dynamics (CFD) and ML will provide standardized, targeted training and rehearsal for interventional procedures in the future. Additionally, researchers have attempted to use AI to identify surgical actions and assess the quality of these actions in relation to surgical outcomes [78,79,80,81]. Similarly, we can apply models to recognize operators’ handling of interventional instruments, establishing deep learning models to standardize techniques and thereby evaluate and guide novices’ actions.

On the other hand, AI-enhanced imaging technology not only reveals finer lesions in radiology but also benefits interventional treatment. Based on preoperative patient images, AI models can highlight lesions in real-time images and improve image quality under a fixed X-ray dose, improving treating quality effectively. The development of remote treatment also benefits from advancements in AI and networking technologies. Current remote procedures, whether operated locally or controlled via a network, face issues with latency and insufficient haptic feedback [82]. Theoretically, by training AI models to recognize images transmitted during treatment, we can predict the behavior of guide wires and catheters. In this case, AI can use methods such as manipulating magnetic fields to generate a counterforce for mitigating the excessive manipulations by the physician. This approach could significantly reduce the risk of mistakes due to image transmission delays, thereby minimizing the risks associated with remote interventions [83,84]. Additionally, when AI detects potentially hazardous operations, such as excessive deformation of the guidewire that could lead to adverse outcomes, it will be able to issue an early warning to alert the operator to avoid dangerous actions.

With the rapid development of various large-scale AI models in recent years, AI technology has demonstrated the potential to enhance productivity in multiple fields. In the medical domain, especially through the application of large language models like ChatGPT, these models have shown decent accuracy in medical knowledge, disease diagnosis, and clinical decision-making tests. By conducting a systematic literature search in PubMed, Scopus, and Web of Science using the search terms “(Chatgpt-4 or Gpt-4) AND (assessment OR tests) AND medicine”, we initially screened 445 related publications. And after removing duplicates and articles that did not align with the preliminary research theme, 104 studies were retained for more in-depth analysis. Studies with overly complex evaluation criteria were further excluded, such as those involving the assessment of consistency between physicians and AI models in responses, as well as studies that required the design of detailed scoring criteria for evaluation. Ultimately, a comprehensive analysis was conducted on 57 studies that specifically tested the accuracy of GPT-4 in answering medically related questions. The aggregated data from these studies indicate that the accuracy of GPT-4 in responding to medical inquiries ranges from 47.4% to 100%, with a median value of 80.35%. This study adheres to the PRISMA guidelines, and all data extraction was independently reviewed by two researchers to ensure the reliability of the findings. Considering ongoing advancements in computational technologies and the expansion of training datasets, an improvement in this accuracy rate can be anticipated.

However, to promote the application of generative language model AI technologies, we must strictly adhere to the principles of explainable artificial intelligence (XAI). In the real world, the use of unexplainable black-box AI applications is irresponsible, especially in sensitive fields such as medicine, finance, and law. Future AI technologies should strive to make decision-making processes more transparent and interpretable. This can be achieved by using simplified models to reduce the complexity of deep learning models, making their decision processes easier to understand, or by using proxy models, that is, using simple, interpretable models (such as decision trees) to approximate the behavior of complex models, thereby helping users understand the decision logic of complex models [85].

Furthermore, the future may see the development of technologies that use one AI model to automatically explain the decision-making processes of another model. At the same time, establishing uniform standards and frameworks for explainability to make XAI implementation more consistent and universal across different fields and applications is also an important direction for development [86]. Similarly, improving legal regulations to encourage cautious application of AI technology and prevent misuse should also be prioritized to avoid irreparable consequences due to immature technology or unethical use.

## 3. Interventional Robotics and AI

With the development of medical and computer technology, we can finally bring to the surface the hidden anxieties of interventional physicians and patients. For patients who choose a minimally invasive treatment, they often do not have the luxury of worrying about radiation damage. For interventional physicians, no amount of protection can avoid long-term accumulation of radiation damage. Since the introduction of interventional robots in the 1980s [15], these seemingly insurmountable obstacles have begun to melt away. By introducing remote control, we can precisely control guide wires and catheters on a console that provides multiple imaging data. With the assistance of AI, lesions that were previously difficult to locate can now be accurately positioned, and hand tremors can be eliminated by adaptive features. Soon, interventional therapy will achieve “minimally invasive” in multiple senses, with efficient, effective, fast, and safe treatment results.

### 3.1. Development Significance of Interventional Robots

Complex and intricate, these words aptly describe the various operations performed on blood vessels during interventional therapy. For an interventional physician, mastering the delicate manipulation of various materials requires extensive practice. Furthermore, long hours of focused operation and the burden of wearing a lead protective suit can quickly deplete a doctor’s energy, affecting the outcome of the operation. Remote-controlled robots can maintain stable operation, reducing fatigue for doctors and offering a level of precision far superior to that of an average physician. By applying the latest computer technology, we can even perform operations that were once unimaginable in traditional interventional treatments. This has the potential to significantly shorten operation times and reduce radiation exposure for both doctors and patients [87].

### 3.2. Existing Interventional Robots

The commercially available vascular interventional robots, including CorPath GRX, Magellan Robotic System, Robocath-R-One, and Hansen Medical Sensei X Robotic Catheter System, have achieved precision that is not inferior to, or even surpassing that of manual operation [88] (see Table 3). The introduction of artificial intelligence technology, such as image recognition (ML and computer vision technology), automatic identification of blood vessels and organs, and assistance in planning operating paths, as well as DL technology, helps doctors automatically detect blood vessel stenosis and thrombosis during operation and provide real-time image guidance, enabling lesion localization and tracking. These technologies improve the accuracy and safety of operation, shorten operation time, and reduce risks of therapy.

Meanwhile, non-vascular interventional robots used for biopsies, tumor ablations, and other purposes have also been launched, utilizing CT, fluoroscopy, and MRI guidance to assist interventional physicians (see Table 4). Overall, these robots have reached or even exceeded the precision of experienced interventional physicians in key areas. However, in dealing with complex individual cases and unique anatomical structures in non-vascular treatments, the role of interventional physicians cannot be completely replaced. Experienced physicians can gain additional insights through tactile feedback, providing more references for treatment. Additionally, compared to intravascular interventional robots, there is less research on non-vascular interventional robots, and the studies generally involve smaller sample sizes. Thus, the reliability of these studies still needs further verification.

Despite multiple clinical trials demonstrating the reliability and efficiency of robot-assisted interventional therapy and achieving or even surpassing the precision of manual operations [89,90], the penetration rate of interventional robots in clinical settings remains low. Interventional robots employ many advanced technologies, such as adaptive learning guidewire control, advanced image fusion and operational path planning functions, and a certain degree of automatic navigation capabilities. However, the maturity and acceptance of these technologies still require time to gradually improve. In some complex cases, over-reliance on new technologies may affect doctors’ experiential judgment. Therefore, in such situations, many experienced doctors may still prefer to use traditional surgical methods. In addition, the high introduction costs and additional learning expenses limit the application of interventional robots. Nevertheless, there is no doubt that intelligent interventional robots represent the future development trend of interventional medicine and still possess tremendous potential for growth.

### 3.3. Development Directions of Interventional Robot

#### 3.3.1. Fully Autonomous Interventional Robots

Currently, most medical robots in the world have adopted some types of artificial intelligence technology for image segmentation or motion control, etc., to reduce repetitive operations for doctors and improve efficiency. However, under the requirements of the FDA, the approved medical robots declare that the device is intended to “assist doctors” or “support doctors in precise operations”, which limits medical robots to a tool role. With the development of AI techniques, this role positioning will change, and robots may become decision-makers and implementers of medical actions, while the role of doctors will be more like a supervisor, reviewing the decisions and operations of robots. For example, in radiation therapy implementing robots, CyberKnife^®^ (Accuray, Sunnyvale, CA, USA) can autonomously track the movement of moving cancer targets [91], adapt to the patient’s breathing and other movements, and use a 6-degree-of-freedom robotic arm to perform radiation therapy on the target. This process is carried out autonomously under the supervision of a doctor, which is an early manifestation of the autonomy of medical robots. Extremely autonomous interventional robots can considerably reduce the repetitiveness of doctors’ operations and help complete catheter and guidewire operation skills that are difficult to achieve with manual operations dealing with complex anatomical structures.

**Figure 1:**
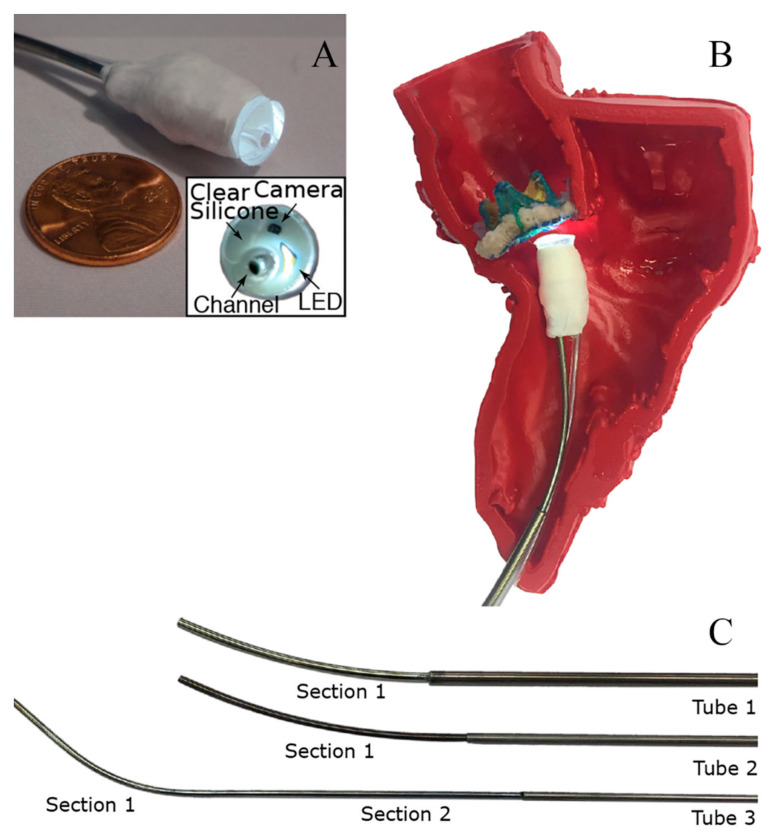
The main structure of the haptic vision robot [92]: (**A**) Haptic vision sensor comprises millimeter-scale camera and LED encased in silicone optical window with working channel for device delivery. Endoscope acts as a combined contact and imaging sensor with optical window displacing blood between camera and tissue during contact. (**B**) Fabricated catheter with haptic vision sensor combined inside 3D printed model. (**C**) Disassembled robotic catheter: 3 pre-curved superelastic tubes. Adapted from G. Fagogenis, M. Mencattelli, Z. Machaidze, B. Rosa, et al., 2019 [92] Copyright © 2019 by The American Association for the Advancement of Science. Used with permission.

G. Fagogenis’s [92] research was inspired by insects and vertebrates using wall-following as an exploratory mechanism under low visibility conditions. The goal is to develop an automated navigation system for minimally invasive surgery to overcome the limitations of imaging techniques such as ultrasound, fluoroscopy, and cardiac angiography. In current clinical practice, force control is mainly achieved through touch control, while catheter positioning relies on manual manipulation under fluoroscopy. The robotic catheter in this study is composed of three structures: The bottom electric drive system, manufacturing conduit with tactile vision sensors, and concentrically combined pre-bent superelastic tubes are the three components (Figure 1). The catheter is equipped with visual-tactile sensors, providing clear images of the catheter tip in contact with cardiac tissue while also identifying the type and force of objects being touched. The researchers also developed a wall-following algorithm that uses the flexibility of the catheter and sensor feedback to adjust the catheter’s position and direction, maintaining low-force contact with the heart tissue and moving along tissue boundaries to reach the target location. The algorithm is divided into continuous contact mode and intermittent contact mode. In addition, the study developed a graphical differentiation technique that can distinguish between blood, ventricular wall tissue, and artificial heart valves. This technique uses OpenCV to detect features in manually marked training images and then mathematically encodes the detected features using LUCID [93] descriptors. Optimal feature representatives are identified using a clustering algorithm (k-means), and feature histograms are constructed. Finally, a support vector machine classifier is used to learn the relationships between feature histograms and their corresponding categories. The automatic navigation software is written in C++ and can alarm the physician when the catheter reaches the target location.

This technology can be applied to operations such as paravalvular leak repair and has the potential to be used in a broader range of operation fields in the future. According to the research results, there is no significant difference in success rates between left ventricular apex to aortic annulus and left ventricular apex to aortic annulus, both exceeding 90%. In terms of annular movement to the target location at the valve ring, the success rate of automatic navigation is 66%, which is lower than the other two methods. As for the time required, the automatic mode takes the longest, but it is within an acceptable range.

If the first barrier for interventional physicians to accept interventional robots is to give up tactile control of guidewires and catheters, then the reliability of artificial intelligence technologies, especially DL and computer vision (CV) technologies, is another concern for doctors. At present, computer vision technology based on CNN has been widely applied in fields such as image classification [94], face recognition, and biometrics recognition [95], showing superior performance compared to traditional CV methods [96], particularly in object detection, tracking, and classification. However, training deep models requires a large amount of image data, and the lack of data may lead to reduced model reliability.

Fully automated interventional robots need excellent target positioning and image segmentation capabilities to identify lesions in complex anatomical structures, choose appropriate paths to reach designated locations, and perform corresponding operations. A classic example is the instrument recognition method based on DL proposed by MIT, which uses an architecture based on deep residual models (U-Net) and labels each pixel of each image in eight videos (each with 255 frames) as either an instrument or background to train the model. In the non-real-time version, the balanced accuracy reached 80.6%, while in the real-time version, the balanced accuracy was 78.2%. Building on such research, Zhao et al. [97] used two different CNNs for real-time multi-tool detection; the first CNN output detection heatmaps indicating the positions of tooltips, and the second one performed bounding box regression on these heatmaps stacked on input Red, Green, Blue (RGB) image frames. The results showed that both detection speed and accuracy improved. As for object detection and tracking, Lee et al. [98] proposed a CNN-based cross-frame real-time recognition and localization model trained on 54 Da Vinci surgical robot operations video segments. It evaluated its performance using the root mean square error and AUC. The results showed that the model could track and locate surgical instruments during surgery and had the potential to replace existing surgical skill assessment methods.

To achieve complete automation control of interventional devices, more than single-center research is required to meet many samples necessary for DL models. For specific operations, it is vital to establish a public DSA image database and formulate unified image standards to promote data sharing and collaboration. This will provide more affluent and more diverse training samples for DL models, helping to improve the accuracy of the trained models and ensuring that serious consequences caused by inaccurate image recognition do not occur during the automation process. In addition, Meta’s semantic segmentation model, the Segment Anything Model, serves as the largest segmentation dataset to date, containing over 11 million licensed and privacy-respecting images with one billion masks [99]. It is designed and trained to be prompt, allowing for zero-shot transfer to new image distribution tasks with segmentation performance comparable to fully supervised models. By prompting with gaze points detected through wearable devices, SAM can continuously segment and track targets, providing new possibilities for developing future CV technologies. In the foreseeable future, training a CV model incorporating a large number of perspective and DSA images will allow physicians to provide prompts for accurate recognition and segmentation of corresponding locations, such as cerebral vascular images, ensuring reliable identification and positioning for automated interventional therapy.

#### 3.3.2. MRI-Guided Interventional Robotics

In contemporary cardiovascular operations, not only is precision required in the treatment, but it is also essential to incorporate basic physiological information such as perfusion, blood flow, and tissue oxygenation during intravascular interventions to make comprehensive treatment decisions. In addition to its ability to complement single-photon emission computed tomography myocardial perfusion imaging in guiding PCI [100], cardiovascular magnetic resonance has the advantage of no ionizing radiation and provides good soft tissue contrast [101]. Therefore, MRI-guided interventional therapy navigation has a promising future. MRI can provide functional information such as blood flow, tissue oxygenation, diffusion, and perfusion, all of which can be fed back to the physician in real-time.

Although MRI provides anatomical and functional information of the target tissue, its spatial and temporal resolution is lower than X-ray fluoroscopy. The most widely used pulse sequence for MR-guided endovascular interventions is the balanced gradient imaging technique, which allows for a relatively high signal-to-noise ratio in short repetition time (TR) sequences. Furthermore, developing stronger, faster gradient magnets, parallel imaging techniques, and alternative k-space trajectories can improve imaging speed [102].

In terms of interventional materials, and in addition to considering the radiofrequency heating effect, MRI guidewires and catheters need to have good visibility [103,104]. To address these concerns, some guidewires and catheters are designed with specialized materials and features. For instance, a certain guidewire [105] is made of a polymer composed of high-strength aramid synthetic fibers (twisted along its longitudinal axis), surrounded by a bending-resistant high-performance polymer composite and a Polytetrafluoroethene layer. Moreover, super paramagnetic iron oxide microparticles embedded in curable resin are incorporated into various parts of the guidewire to enhance visibility.

Currently, there is no comprehensive MRI vascular intervention robotic system. However, to address the issue of MRI noise interference and to introduce remote operation and intelligent assistance features, the development of MRI primary–subordinate robots is becoming a research hotspot.

The hydraulic-driven vascular intervention robot system developed by Li et al. [106] suffers from motion lag due to gear clearance caused by the gear-rack mechanism used to convert motion types. Abdelaziz et al. [107] developed a pneumatically-driven vascular intervention delivery mechanism; compared to hydraulic drives, gases have poorer compressibility, resulting in unstable transmission and slow response. Tavallaei et al. [108] proposed a magnetic resonance-compatible remote catheter navigation system with a complex structure and numerous intermediate transmission mechanisms, making it challenging to achieve high-precision motion control. Liu et al. proposed a three-dimensional motion model for a controllable ablation catheter system with energized micro-coils embedded in the catheter, generating magnetic forces under an MRI scanner’s magnetic field for deflection movement. However, this drive method can only be used for single bending deformation of the catheter and cannot achieve axial and rotational movements, making it unsuitable for vascular interventional operation.

**Figure 2:**
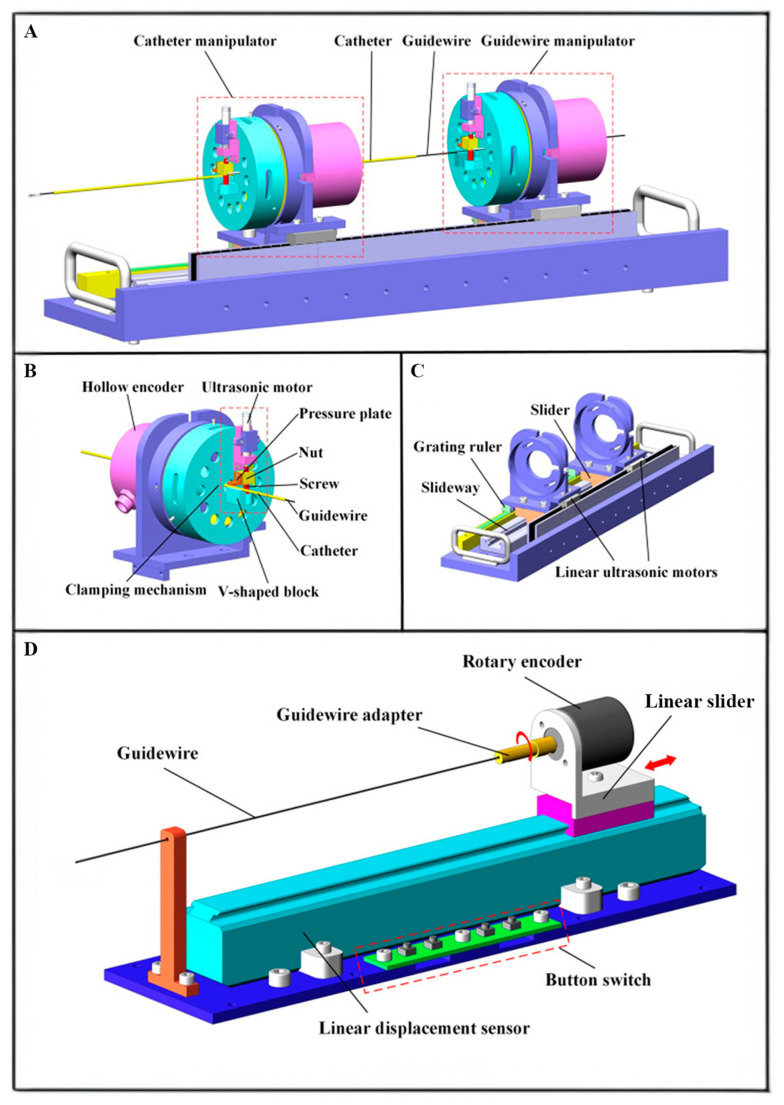
The main components of the primary robot and the subordinate robot in Lu Qing’s research: (**A**) The subordinate robot (**B**); Detailed structure of hollow mechanism; (**C**) The structure of the linear motion platform; (**D**) Main components of the primary robot. AdaptedA Novel Remote-Controlled Vascular Interventional Robotic System Based on Hollow Ultrasonic Motor” by Lu Qing, et al., 2022 [109]. CC BY 4.0. Used with permission.

Lu Qing et al. [109] introduced a remote-controlled vascular intervention robot for MRI-guided navigation. The remote vascular intervention robot uses a traveling wave hollow ultrasonic motor (HUM) to perform complex vascular interventional treatments in an MRI environment. By employing an adaptive genetic algorithm, the system provides better wave quality and a larger amplitude, which benefits driving efficiency and precision. The system adopts a primary–subordinate architecture, in which the surgeon manipulates the guidewire’s rotational and linear movements (Figure 2). These movements are captured and transmitted to the control unit, which drives the subordinate robot to control the guidewire and catheter movement within the blood vessels. The position information of the guidewire and catheter is detected via MRI, and the surgeon uses the imaging as visual feedback for the subsequent movement. This system uses a hollow mechanism HUM, a linear platform, and a linear ultrasonic motor to drive the guidewire and catheter directly, simplifying the structure and improving response sensitivity. In addition, the system introduces an adaptive genetic algorithm to optimize the HUM stator, allowing it to generate ideal traveling waves. The optimized stator’s interference mode is far from the working mode, resulting in better wave quality. As a result, the system can perform interventions under MRI, providing more accurate intravascular images and operational precision.

In addition to the application scenarios of intravascular interventions, MRI-guided non-vascular interventional robots are undergoing trials in various clinical applications. For example, the robot system for MRI-guided prostate punctures can accurately locate, puncture, and ablate lesions, significantly improving puncture accuracy compared to manual operation and providing soft tissue information for ablation, offering a good reference for whether the ablation boundary meets requirements. At present, several MRI-safe or MRI-compatible robot products have been developed. As the first FDA-approved MR safe robot device, MrBot is made entirely of non-conductive and non-magnetic materials and has a 6-degree-of-freedom pneumatic drive. In human puncture biopsy experiments, it achieves high accuracy (3D accuracy of 2.97 mm and normal plane accuracy of 2.55 mm) [110], meeting the 5 mm accuracy requirement for prostate puncture. Although this direct MRI navigation method avoids the drawback of not being able to verify targeting in MRI in the MRI- transrectal ultrasound fusion approach, the current issue of prolonged operation time still needs to be addressed. This is expected to improve in the future.

#### 3.3.3. Magnetic Controlled Interventional Robotics

In traditional endovascular treatment, precise manipulation of instruments, including catheters and guidewires, is crucial for the success of the operation. However, the accurate control of guidewires is challenged by their fine and flexible structure, unpredictable interactions with tissue, and nonlinear operational forces [111]. In the field of neurointervention, the use of microcatheters is limited to lesions that can be safely reached, making precise and effective delivery of therapeutic agents to lesions located in the distal cerebral cortex difficult. Also, exposure to radiation during interventional procedures poses a significant health threat to physicians. To address these challenges, some researchers have turned their attention to the study of magnetic control interventional robot devices. These millimeter/micrometer scale medical devices can enter distal lesion vessels and perform interventional treatment operations such as drug release and vessel opening under magnetic field control. Based on existing research, magnetic control robot devices can be divided into tethered and untethered types. Tethered magnetic robots, generally based on guidewire or catheter structures, can be manipulated and controlled by electromotive force and safely removed from within the vessel through the tail-end guidewire during surgery [112,113,114]. However, in some cases of treating distal curved vessels, tethered magnetic robots may lose control or break due to the contact force exerted by curved vessels. During interventional treatment, some tethered magnetic robots controlled by rotating electromotive force may twist and vibrate, damaging the vessels. Conversely, untethered magnetic robots (UMRs) can precisely navigate complex paths under magnetic field control, releasing local medications or opening blocked vessels by designing corresponding forms of UMRs to fit the target vessel structure.

Wanelg Tianlu et al. designed a stent-type soft UMR device, small enough to adapt to vascular morphology and reach distal vessels that traditional interventional treatments cannot [115]. It has self-anchoring capabilities, remaining positioned in lumens as small as 1 mm in diameter and in vessels with a minimum curvature radius of 3 mm, resisting the effects of blood flow at speeds up to 26 cm/s and 80 pulse movements per minute. During vascular intervention, the device can release medications like tissue plasminogen activator (tPA) on demand at the target location and can act as a diversion device to reduce blood flow within an aneurysm or to targeted branch vessels. This device offers a new minimally invasive targeted treatment method for distal and twisted vascular areas, including AIS, aneurysms, cerebral arteriovenous malformation (CAVM), and brain tumors. In simulated lumen systems and ex vivo pig coronary arteries, the device has shown self-anchoring capabilities and successful navigation to the target distal area under magnetic control while exerting less force on vessel walls compared to standard neurointerventional catheters. The device utilizes radiofrequency heating of a shape memory polymer (SMP) box to release medication, achieving targeted drug application and minimizing unnecessary complications.

In practice, the current propulsion force of UMRs is low, making it impractical to anchor effectively in large luminal vessels and under rapid blood flow, posing the risk of UMR loss and unpredictable outcomes. To address this, Park et al. [116] attempted to transport UMRs into relatively safe vessels using a magnetic assembly structure made of a permanent magnet at the end of the catheter. However, this could not avoid the interference from the additional magnetic field generated. Junchi Sa et al. designed a separable and recombinable magnetic robot (SRMR), connecting the transport device and UMR with a spiral design [116]. When near the target vessel, they used rotating EMF and tunnels to separate the two and performed operations like opening occluded vessels with a drill tip under magnetic control. They successfully demonstrated the separation and recombination of UMRs, opened occluded pseudo-vessels, performed local drug delivery in simulated devices, and performed maneuvers such as steering, contrast agent delivery, device separation, movement, and recombination in the femoral artery of mini pigs with SRMR.

Overall, to ensure that magnetic control robots can meet the functional and safety requirements for use within the human body, their design should consider the following key factors:

1. Translating Clinical Needs into Design Specifications: The design process should fully consider specific clinical requirements, such as adjusting the robot’s maximum outer diameter based on the average diameter of the target blood vessel, to ensure that blood flow is not obstructed, thereby avoiding reduced blood flow or stasis.
2. Complex Pathway Navigation Ability: The robot should be capable of flexibly navigating through the tortuous and complex pathways of the cardiovascular system, ensuring that it does not damage the blood vessel structure, especially in vessels with arteriosclerosis.
3. High Sensitivity and Rapid Response to Magnetic Fields: The robot should be highly sensitive to magnetic fields and capable of rapid responses, enabling intuitive and logical remote operations.
4. Good Biocompatibility: Ensure that the robot’s materials and design meet biomedical standards to minimize potential risks to the human body.
5. Simplified Recovery and High Reliability: The design should simplify the robot’s recovery process and ensure its high reliability during operations.
6. Self-Anchoring Ability: Even in the absence of magnetic field control, the robot should maintain its stability, avoiding displacement or unnecessary damage.

Currently, magnetic control robot devices come in various forms, with many prototypes showing potential for application. With the continuous progress in materials science and automation control technology, we anticipate the emergence of mature magnetic control robot devices in the market. These devices will serve as vital tools for endovascular intervention, bringing revolutionary improvements to traditional treatment methods and significantly enhancing the precision and safety of treatments.

## 4. Discussion and Outlook

**Figure 3:**
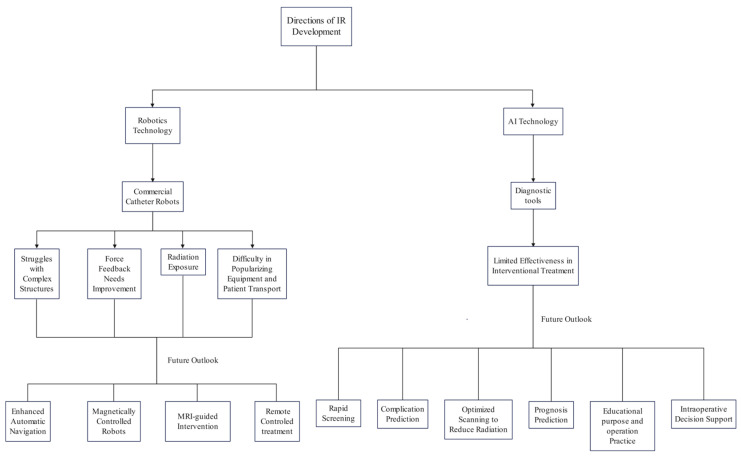
Previously cited Shortcomings and Development Directions of AI and Robotic Technologies in IR.

AI and robotic systems have been transforming medical practice, enabling rapid and efficient diagnosis and treatment, improving diagnostic accuracy, and facilitating precise and safe interventions. Their impact is already evident in current clinical interventional treatments, but the immaturity of these technologies and insufficient clinical recognition limits their application. This study reviews the limitations of existing technologies and discusses the future directions of IR based on previous research (see Figure 3). The application of AI and robotic technology in clinical settings is not yet widespread, and the challenges extend beyond just the technological aspects. The high costs associated with research and development, coupled with the expensive pricing of commercial equipment, necessitate substantial investment from pioneers in the field to gain acceptance from patients and healthcare providers. Most AI applications in IR are still in the initial research and clinical trial phases. However, the potential seen in a few commercialized diagnostic and treatment products highlights advancements unattainable with traditional interventional methods. For instance, interventional treatment simulation systems can accurately replicate human anatomical structures and synchronize with DSA images, providing a realistic tactile experience. This is particularly beneficial for training new doctors on high-risk procedures such as embolization of aneurysms. Given time, modern interventional radiology is poised to alleviate patient suffering through more intelligent, minimally invasive, and safer methods.

However, despite significant advances in the integration of AI, robotics, and IR, many uncertainties still need to be addressed. AI is not flawless and cannot fully replace human judgment and decision-making. In fact, AI should be seen as a complement and enhancement to human intelligence. Just like drugs and surgical tools, AI is a tool in clinical treatment that helps alleviate patients’ ailments. Therefore, in clinical practice, the use of AI should be approached with caution and a critical attitude. Although AI’s errors may decrease with ongoing technological iterations, we must still be vigilant about its potential risks and ensure its applications do not harm patient interests. With regard to robotics and automation technologies, we must also maintain a cautious approach, consistently defining them as tools to assist physicians and ensuring they operate under continuous supervision.

While envisioning a bright future for interventional radiology, it is essential to acknowledge that, currently, the public’s understanding of interventional treatments remains superficial. According to an anonymous survey conducted on Amazon’s Mechanical Turk crowdsourcing platform, the majority of respondents (39.8%) mistakenly believe that interventional radiology is an independent discipline rather than a treatment modality [117]. Despite this, after learning about interventional treatments, the vast majority (92%) expressed willingness to undergo such therapies. To advance new interventional techniques and promote the clinical application of AI and robotics within this field, establishing a solid foundation of public education is paramount. This is crucial not only for patients but also for clinicians who are not specialists in this area. It is vital not to overstate the benefits to patients and to accurately present the advantages and potential risks of interventional treatments and the AI and robotics technologies that may be employed during procedures. Ensuring informed consent, continuously building a good reputation, and gaining the trust of both clinical departments and patients are essential for the healthy development of interventional radiology in the future.

Additionally, practitioners should remain vigilant about the uncertainties inherent in these new technologies, regardless of how advanced these applications may be. For instance, AI image recognition can be easily disrupted by customized adversarial images, leading to incorrect identification of shapes or structures [118]. While such adversarial patterns are unlikely to occur naturally if utilized for criminal purposes, any oversight by the practitioner undoubtedly opens the last line of defense for criminals. Furthermore, robotic technologies and automation may struggle to effectively respond to sudden or significant movements by patients. Merely pausing the procedure may not always be the safest option. In such cases, the ability of the interventional physician to respond effectively and the patient’s trust in new technology are of utmost importance.

To delve deeper into the obstacles to the application of AI and robotics technologies and the directions we need to strive toward, we can start with the data dependency and model training of AI. We know that the output of any AI tool is highly dependent on the quantity and quality of input data. The performance of DL increases with the amount of data. If the data quantity is limited, the results obtained may not be as good as expected because it requires a large amount of data to understand the patterns contained in it. In research, image datasets such as DSA are pre-screened to exclude some low-quality images, such as motion artifacts or excessive noise, to ensure data quality. However, such screening may also lead to a decline in performance in actual use, as unfiltered data may be closer to real clinical situations, thus better reflecting clinical interference, noise, and other issues. Therefore, augmenting the dataset is crucial, as only a large amount of data can result in a sufficient representation of disease characteristics and inter-individual variability. In addition, only prospective studies can genuinely analyze the performance of trained AI models in clinical practice. Therefore, a large amount of multi-center training data is needed to improve classification performance and generalizability. Many studies remain within single-center retrospective data research, and the trained models are not universally applicable to other data. The ideal mode should conduct prospective continuous research tests on datasets and carry out additional optimization and integration of research modules to seamlessly embed these modules into clinical workflows.

Secondly, scanning costs and patient privacy issues should also be considered. Providing AI systems with a large amount of accurately registered, segmented, and labeled data is expensive, and obtaining high-quality medical image annotations may even be more costly than obtaining medical images themselves. Furthermore, the widespread use of AI-based intelligent and autonomous systems in the future may increase the risk of systemic errors and highlight complex ethical and social issues. Security and ethical issues are a global concern, and data privacy has always been a theme of the European General Data Protection Regulation (GDPR) [119]. Doctors and other personnel who come into contact with patient data should protect patient information as much as possible, which may be even more important than protecting data for healthy individuals [120]. We need to find a balance between patient privacy, ethics, and AI company partners on data protection issues [121]. This will improve doctors’ job satisfaction, enhance doctor-patient relationships, and increase the quality of healthcare services. Developing commercial interventional robotics hardware and software requires a substantial amount of clinical data and experimentation. This process not only involves technical challenges but also necessitates the consideration of legal requirements related to patient privacy and data protection. Even when approved for market, the safety measures of robotic systems should be regularly assessed to prevent potential systemic errors and address any resulting ethical and social issues.

Lastly, we must still interpret machine decisions and predictions to demonstrate their reliability. At present, we know very little about many machine decisions, and the black-box nature of DL still needs to be solved. Due to the need for more interpretability, AI tools based on DL for predicting risk or making differential diagnoses may encounter problems. Eliminating the black box in AI is an active research area aiming to reduce uncertainty about how decisions are reached [122,123]. With a deep understanding of the processes behind a given output, it may be easier to determine whether clinical decisions to respect the algorithm are feasible. For example, how much weight should the doctor assign to the algorithm’s diagnosis in cases of diagnostic disagreement? And when mistakes are made following AI decisions, should the legal liability fall on the AI tool provider or the doctor? Legal frameworks for the development of standards in AI and autonomous robotic operation are still evolving, and it will likely take some time before globally accepted standards are established [123,124]. In the foreseeable future, AI’s ultimate responsibility and accountability will still be borne by its human designers and operators [125]. Therefore, we should focus on developing XAI methods to assist clinical practitioners in their medical practice. As high-risk decisions are intertwined with medicine, there is growing concern that these black boxes may harbor biases somehow, and overlooking such biases could have far-reaching consequences. XAI will become increasingly important, and the key to success lies in multidisciplinary approaches [126]. We need to encourage and promote data-driven, mathematical, and technologically-based medical education, while training imaging professionals to become AI experts to understand the needs and challenges of clinical settings.

## 5. Conclusions

The development and refinement of AI and robotics technologies signify the advent of a new era in IR. The most significant innovation in this field is the capability of magnetically controlled interventional robots, remote-controlled robotic technologies, MRI-guided interventional robots, and highly automated catheter robots to reduce radiation exposure to patients and medical staff during interventional treatments. Additionally, the application of AI in interventional therapy is no longer confined to pre-operative imaging diagnosis but extends throughout the entire treatment process, optimizing the quality of interventions from multiple aspects. Our research indicates that AI and robotics hold substantial potential in clinical interventional applications; the application of AI and robotics in interventional radiology assists physicians in making faster, more accurate, and cost-effective decisions in diagnosis and treatment management, thereby enhancing patient satisfaction and trust. Simultaneously, it is crucial to remain vigilant against the potential misuse of AI and robotics and to strive to develop interpretable AI models and standardized robotic technology usage protocols to prevent ethical breaches and operational errors, ensuring the responsible and safe use of these advanced technologies. Currently, the application of AI and robotics in interventional procedures is still in its nascent stages; however, even at this early juncture, the prospects for a promising future are distinctly visible.

## Figures and Tables

**Table 3:** Commercial intravascular interventional robots.

Product Name	Company Name	DOF	Application Scope	Feedback	Features	Navigation
Corpath GRX	Corindus Vascular Robotics	5 DOF	Cardiovascular, Peripheral, Neuro	Haptic Feedback	High precision (up to 0.1 mm), Intravascular Endoscopy	Digital geometric imaging and Machine Vision, Dedicated software for automation assistance
Magellan	Hansen Medical	7 DOF	Cardio-vascular, Neuro-vascular, Peripheral	Force Feedback	C-Arm 3D Scanning Localization	Magnetic Field Sensing and Machine Vision
Robocath R-One	Robocath	N/A	Cardiovascular	Bidirectional Force Feedback	Enhanced Motion (Continuous Rotation, etc.)	AI algorithms assist in real-time generation of 3D images, Machine Vision
Sensei X2 Robotic Catheter System	Hansen Medical	3 DOF	Cardio-vascular	Haptic Feedback	Automatic detection and tracking of heart position and motion	Digital geometric imaging and Machine Vision
Amigo	Catheter Precision	3 DOF	Cardio-vascular	Haptic Feedback	Open catheter architecture	-

DOF: Degrees of Freedom.

**Table 4:** Commercial non-vascular interventional robots.

Product Name	Company Name	DOF	Application Scope	Imaging
AcuBot	Hopkins	6 DOF	Biopsy, drainage, Tumour ablation, Vertebroplasty	Fluoroscopy, CT
B-Rob II	ARC Seibersdorf Research	7 DOF	Biopsies	CT, US
iSYS1	Medizintechnik	4 DOF	Biopsy	Fluoroscopy, CT,
INNOMOTION	Innomedic	6 DOF	Biopsy, Tumour ablation, Drainage	CT, MRI
EPIONE	Quantum Surgical	6 DOF	Tumour ablation	CT

## Data Availability

Data availability is not applicable to this article as no new data were created or analyzed in this study.
